# Liposomes to Target Peripheral Neurons and Schwann Cells

**DOI:** 10.1371/journal.pone.0078724

**Published:** 2013-11-11

**Authors:** Sooyeon Lee, Ana Tari Ashizawa, Kwang Sik Kim, Darin J. Falk, Lucia Notterpek

**Affiliations:** 1 Department of Neuroscience, College of Medicine, University of Florida, Gainesville, Florida, United States of America; 2 Department of Neurology, College of Medicine, University of Florida, Gainesville, Florida, United States of America; 3 Eudowood Division of Pediatric Infectious Diseases, Johns Hopkins Children's Center, Baltimore, Maryland, United States of America; 4 Department of Pediatrics, College of Medicine, University of Florida, Gainesville, Florida, United States of America; NHLBI, NIH, United States of America

## Abstract

While a wealth of literature for tissue-specific liposomes is emerging, optimal formulations to target the cells of the peripheral nervous system (PNS) are lacking. In this study, we asked whether a novel formulation of phospholipid-based liposomes could be optimized for preferential uptake by microvascular endothelia, peripheral neurons and Schwann cells. Here, we report a unique formulation consisting of a phospholipid, a polymer surfactant and cholesterol that result in enhanced uptake by targeted cells. Using fluorescently labeled liposomes, we followed particle internalization and trafficking through a distinct route from dextran and escape from degradative compartments, such as lysosomes. In cultures of non-myelinating Schwann cells, liposomes associate with the lipid raft marker Cholera toxin, and their internalization is inhibited by disruption of lipid rafts or actin polymerization. In contrast, pharmacological inhibition of clathrin-mediated endocytosis does not significantly impact liposome entry. To evaluate the efficacy of liposome targeting in tissues, we utilized myelinating explant cultures of dorsal root ganglia and isolated diaphragm preparations, both of which contain peripheral neurons and myelinating Schwann cells. In these models, we detected preferential liposome uptake into neurons and glial cells in comparison to surrounding muscle tissue. Furthermore, *in vivo* liposome administration by intramuscular or intravenous injection confirmed that the particles were delivered to myelinated peripheral nerves. Within the CNS, we detected the liposomes in choroid epithelium, but not in myelinated white matter regions or in brain parenchyma. The described nanoparticles represent a novel neurophilic delivery vehicle for targeting small therapeutic compounds, biological molecules, or imaging reagents into peripheral neurons and Schwann cells, and provide a major advancement toward developing effective therapies for peripheral neuropathies.

## Introduction

Targeted delivery of therapeutic compounds to specific cell types is critical in developing effective and safe treatments for neurodegenerative disorders. This challenge is particularly great when designing reagents for diseases of the peripheral nervous system (PNS), such as hereditary motor and sensory neuropathies (HMSNs). Because of the extensive lengths of peripheral nerves, effective approaches will likely require systemic drug administration. Lipid nanoparticles are attractive options for delivery to the PNS as they can be optimized for specific cell types, have shown low toxicity *in vivo*, and can carry hydrophobic and hydrophilic molecules. Despite the progress in engineering tissue-specific liposomes, information about optimal formulations for Schwann cells or peripheral neurons is lacking. As liposomes are known to enter cells primarily via the endocytic pathway [1], [2], and endocytosis is central for neuronal activity [3], [4], [5], we chose liposomes for further development. While the role of endocytosis in Schwann cells is less understood, recent studies demonstrate the involvement of the endocytic pathway in membrane remodeling during myelination [6], [7]. In addition, remyelination of regenerated peripheral nerves is dependent on an intact endosomal-lysosomal system [8].

Endocytosis can occur through clathrin-dependent and independent mechanisms, including caveolae, or lipid raft mediated pathways [9]. We aimed to optimize liposomes for entry through clathrin-independent mechanisms that have been shown to support intracellular drug delivery and endosomal escape [10]. Dioleoyl-phosphatidylcholine (DOPC) was used to construct the liposomes because DOPC is biocompatible, highly versatile, and easy to manipulate. To facilitate particle delivery through the blood-nerve barrier (BNB), we added Poloxamer 188 (P188), a non-ionic emulsifier composed of 30 hydrophobic polypropylene oxide units flanked by 75 hydrophilic polyethylene oxide units on each side. Reports suggest that P188 facilitates the transport of macromolecules across the endothelial cells of the blood brain barrier [11], [12]; therefore it could be beneficial for liposome transit through the blood-nerve barrier as well. We also added cholesterol (Chol) to the formulation to enhance internalization by PNS cells, since neural membranes, particularly myelinating glial membranes, contain high levels of cholesterol [13].

Using in vitro and in vivo model systems, here we demonstrate that our novel liposome formulation composed of DOPC/P188/Chol is avidly taken up by microvascular endothelia, choroid epithelia, peripheral neurons, and myelinating and non-myelinating Schwann cells. Because of the suitability of these liposomes for local injections as well as systemic administration they represent a versatile delivery vehicle for therapeutic applications.

## Materials and Methods

### Ethics Statement

All animal studies were performed in accordance with the Standards for the Care and Use of Laboratory Animals – U. S. National Research Council, Statement of Compliance A5023-01, and approved by University of Florida Institutional Animal Care and Use Committee (IACUC).

### Animals

Rats (Harlan Laboratories) and C57/BL6 mice (Jackson Laboratories) were housed under SPF conditions at the University of Florida McKnight Brain Institute animal facility. All animals were euthanized by CO_2_ asphyxiation prior to tissue collection.

### Cell culture

Human neuroblastoma SH-SY5Y cells and murine embryonic NIH-3T3 fibroblasts (American Type Culture Collection) were grown in Dulbecco's modified Eagles medium (DMEM) supplemented with 10% fetal bovine serum (FBS; Hyclone). Brain microvascular endothelial cells (BMECs) were cultured in Medium-199 (Lonza) supplemented with 10% FBS and 10% NuSerum [14]. Primary Schwann cells were prepared from the sciatic nerves of neonatal rat pups as described [15]. Myelinating dorsal root ganglion (DRG) explants were established from embryonic days 12–15 mouse pups [16]. Murine C2C12 myoblasts (American Type Culture Collection) were cultured in growth medium (DMEM supplemented with 10% FBS, 1% penicillin/streptomycin, and 0.1% fungizone) until cells reached 80–90% confluence, at which point myoblast differentiation was induced by incubation in DMEM supplemented with 2% heat-inactivated horse serum, 1% penicillin/streptomycin, and 0.1% fungizone.

### Liposome preparation, ultrastructural analyses, and particle sizing

Liposomes were prepared by the lyophilization method [17], [18], [19]. DOPC and Chol were purchased from Avanti Polar Lipids, Inc. P188 (Sigma-Aldrich Chem.) and Chol was added to DOPC in weight ratios ranging from 0 to 50 percent in the presence of excess tertiary butanol (Fisher). Fluorescent phospholipids, 1,2-dioleoyl-sn-glycero-3-phosphoethanolamine-N-carboxyfluorescein (CF; Avanti Polar Lipids, Inc.) or Cyanine 5-N-hydroxysuccinimide ester (Cy5; Lumiprobe Corp.) were added to the lipid mixture at 2–10 mole percent. Lipid mixtures were frozen at −80°C overnight and lyophilized with a FreeZone 2.5 lyophilizer (Labconco), followed by storage for up to two months at −20°C. DOPC/P188/Chol liposomes reconstituted in 0.9% NaCl were whole mounted on degaussed nickel mesh grids, stained with 1% uranyl acetate and examined with a Hitachi H-7000 transmission electron microscope (Hitachi High Technologies America, Inc.). Digital images were acquired with a Veleta 2k×2k camera and iTEM software (Olympus Soft-Imaging Solutions Corp). Particle size was measured by Nanotrac (Microtrac), which uses dynamic light scattering to determine nano size particulates.

### Fluorescence analysis by flow cytometry

Cells incubated with fluorescent liposomes (200 µM) for 24 h were trypsinized and suspended in 0.1 M phosphate buffered saline (PBS) containing 2% FBS. Cell suspensions were analyzed on an Accuri C6 flow cytometer (BD Biosciences) with a minimum cell count of 10,000 cells per sample. Experiments were repeated at least three independent times using triplicate samples.

### Cell Viability Assay

Cell viability was analyzed by trypan blue dye exclusion. Cells plated in 12-well dishes were dissociated with 0.05% Trypsin and resuspended in 0.4% trypan blue (Invitrogen). Using a hemocytometer, the total number of cells and cells stained with trypan blue were recorded. Cell viability was determined as the number of viable cells (trypan blue negative) divided by the total number of cells. Each condition was analyzed in triplicates.

### Liposome uptake in BMEC, Schwann and C2C12 cells

BMECs, Schwann and C2C12 cells were incubated with 200 µM liposomes for 8–24 h at 37°C. Cells were rinsed in 0.1 M PBS, fixed with 4% paraformaldehyde (Electron Microscopy Sciences) for 10 min, stained with Hoechst dye (Invitrogen) and imaged immediately. LysoTracker® Red DND-99, pHrodo™ Red dextran (10,000 MW), or BODIPY®-493/530 (all purchased from Invitrogen) were either co-incubated with the liposomes (dextran) or added for the last 30 min at 37°C prior to fixation (LysoTracker, BODIPY 493/503). Promazine (0.5 µM), cytochalasin D (0.1 µg/mL) and methyl-β-cyclodextrin (MBCD; 4–10 mM) were purchased from Sigma and co-incubated with liposomes for 8 h at 37°C. For cell surface liposome binding and lipid raft staining, Schwann cells in normal growth media were rinsed twice in cold 0.1 M PBS first and co-incubated with liposomes (2 mM) and Alexa Fluor®-594-Cholera toxin subunit β (Ctx-β; 20 µg/ml) (Invitrogen) diluted in 0.1 M PBS with 0.1 M Mg_2_SO_4_ for 30 min on ice. Samples were rinsed and counterstained with Hoechst dye prior to imaging. All liposome uptake experiments were repeated at least two to three independent times using triplicate samples.

### Liposome uptake in tissue explants

For myelinating dorsal root ganglia (DRG) explants, DOPC/P188/Chol liposomes were reconstituted to 200 µM in normal culture medium and applied for 24–72 h in 37°C at 18 days *in vitro*. Freshly-isolated diaphragms from 4–8 week-old C57/BL6 mice were cultured with modifications [20]. Briefly, diaphragms were dissected into cold artificial cerebral spinal fluid (ACSF) containing Alexa Fluor® 594 conjugated α-Bungarotoxin (α-Btx; 1 μM) (Invitrogen). Tissues were then transferred to fresh ACSF containing fluorescent DOPC/P188/Chol (0.2–2 mM), and incubated at 37°C for 4–8 h. Samples were rinsed twice with 0.1 M PBS and fixed with 4% paraformaldehyde for 10 min (DRGs) or 60 min (diaphragms) at room temperature, rinsed with 0.1 M PBS and counterstained with Hoechst dye. These ex vivo experiments were repeated at least three independent times using different batches of liposomes.

### Liposome administration in vivo and tissue processing

Particles were reconstituted to 20 mM alone or with Alexa-594-dextran (10,000 MW, 2%) in sterile saline (0.9% NaCl) and injected into the foot pad (10 μL) (n = 4 mice) or the tail vein (150 μL) (n = 6 mice) of 2-month old mice. After 4 or 24 h, mice were euthanized by CO_2_ asphyxiation and tissues were dissected into 4% paraformaldehyde in PBS and fixed for 3 h at room temperature, followed by cryoprotection in 30% sucrose in 0.1 M PBS for 24 h at 4°C. Cryosections (20 µm thickness) were rinsed twice in 0.1 M PBS, and stained with Hoechst dye (Invitrogen).

### Optical imaging

Tissues or coverslips were mounted with Prolong Antifade solution (Invitrogen). Fluorescent images were acquired using a Leica spinning disk confocal microscope TCS SP2 equipped with a Hamamatsu camera, or a Nikon Eclipse 800 microscope equipped with a SPOT digital camera. Images were resized for figures using Adobe Photoshop CS5. To analyze liposome fluorescence in Schwann cells and myotubes, fluorescence intensity was measured in over 200 individual cells, using 40X images obtained with equal acquisition parameters and ImageJ software (NIH). Corrected total cell-associated fluorescence was determined by dividing the integrated mode pixel intensity by the cell area. The values were exported to MS Excel and normalized to the cell fluoresce of the DMSO-treated control samples, which were set to one. For quantification of liposome fluorescence in liver and kidney, the integrated pixel intensity was measured in 10X images obtained with equal acquisition settings, with 2–3 animals per condition and 8 images per animal.

### Statistical analyses

For each independent experiment done in triplicates, raw data were exported to MS excel to calculate the averages, standard deviation, and the standard error of the means (SEM). Statistical significance was determined using an unpaired 2-tailed Student's t-test.

## Results

### Cholesterol enhances liposome uptake in neural cells

**Figure 1:**
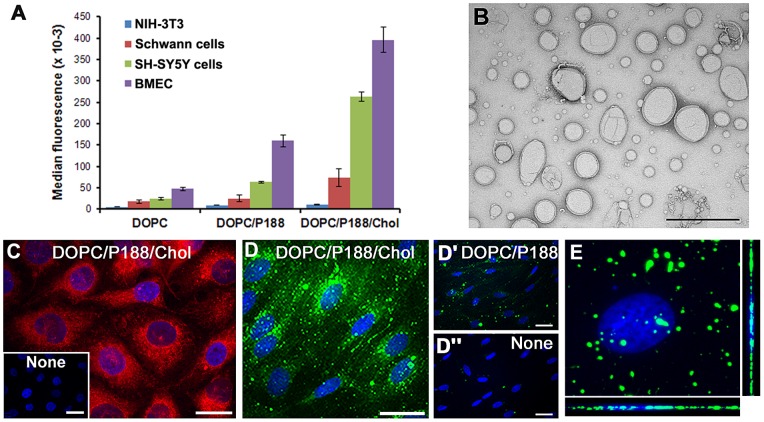
Cholesterol enhances uptake of fluorescent liposomes. Quantification of CF-liposome uptake by flow cytometry in NIH-3T3 fibroblasts, rat Schwann cells, human SH-SY5Y cells and human BMECs (A). Uptake is reported as median fluorescence and expressed in arbitrary units (AU). Values represent means ± std. Whole-mount negative staining electron micrograph of DOPC/P188/Chol liposomes (B). Scale bar, 2 µm. Representative image of DOPC/P188/Chol liposome (red) uptake in BMECs (C). Cells without liposomes are shown in inset. Representative image of DOPC/P188/Chol liposomes in rat Schwann cells (D) compared to DOPC/P188 (D') or untreated (D") cultures. Confocal z-stack image of a Schwann cell after 8h DOPC/P188/Chol liposome (green) uptake (E). The orthogonal y-z and x-z planes are shown on the right and at the bottom, respectively. Nuclei are stained with Hoechst dye and shown in blue (C–E). Scale bars, 20 µm (C–D").

Using flow cytometry, we measured changes in fluorescent DOPC liposome uptake by the addition of P188 alone or with cholesterol (Chol), using NIH-3T3 fibroblasts, human BMECs, human SH-SY5Y neurons, and non-myelinating rat Schwann cells (Figure 1A). The NIH-3T3 fibroblasts served as a representative non-neural cell line. The addition of P188 modestly increased liposome uptake by all neural cells compared to DOPC alone, while the addition of Chol enhanced uptake by 5-, 11- and 8-fold for Schwann cells, SH-SY5Ys and BMECs, respectively. In contrast, we detected little uptake of DOPC liposomes by the NIH-3T3 fibroblasts, which remained low after the addition of P188 and Chol (Figure 1A). These data indicate that the composition of liposomes has a significant impact on their uptake by neural cells and is enhanced by the addition of cholesterol. Of note, brain-derived endothelial cells are particularly active in DOPC/P188/Chol liposome uptake, suggesting that these particles are likely to enter vascular endothelia by systemic administration *in vivo.*

Next, we examined the ultrastructural features of DOPC/P188/Chol liposomes by transmission electron microscopy (Figure 1B). The liposomes are individual spheroid structures, with heterogeneity in size. Measurements using the Nanotrac particle size analyzer indicate that approximately 60% of the liposomes are smaller than 200 nm, making them appropriate as cargo for endocytosis. To confirm that cells internalize the particles, we examined fluorescently labeled liposome uptake by BMECs (Figure 1C) and Schwann cells (Figure 1D and E) using confocal microscopy. After 8 h incubation, internalized fluorescent liposomes were observed in both BMECs and Schwann cells, with diffuse and vesicular pattern in the endothelial cells, and mostly vesicular, punctate distribution in glia. In agreement with the flow cytometry data (Figure 1A), the fluorescent signal associated with the BMEC cultures is brighter, as compared to Schwann cells. To substantiate the internalization of the particles, we obtained confocal z-stack images on Schwann cells after 8 h incubation with DOPC/P188/Chol liposomes. As shown on the orthogonal views along the xz and yz planes, the fluorescent punctate particles are detected at the same plane as the nucleus, indicating intracellular localization (Figure 1E).

### Characterization of cholesterol-enriched liposome uptake by Schwann cells

**Figure 2:**
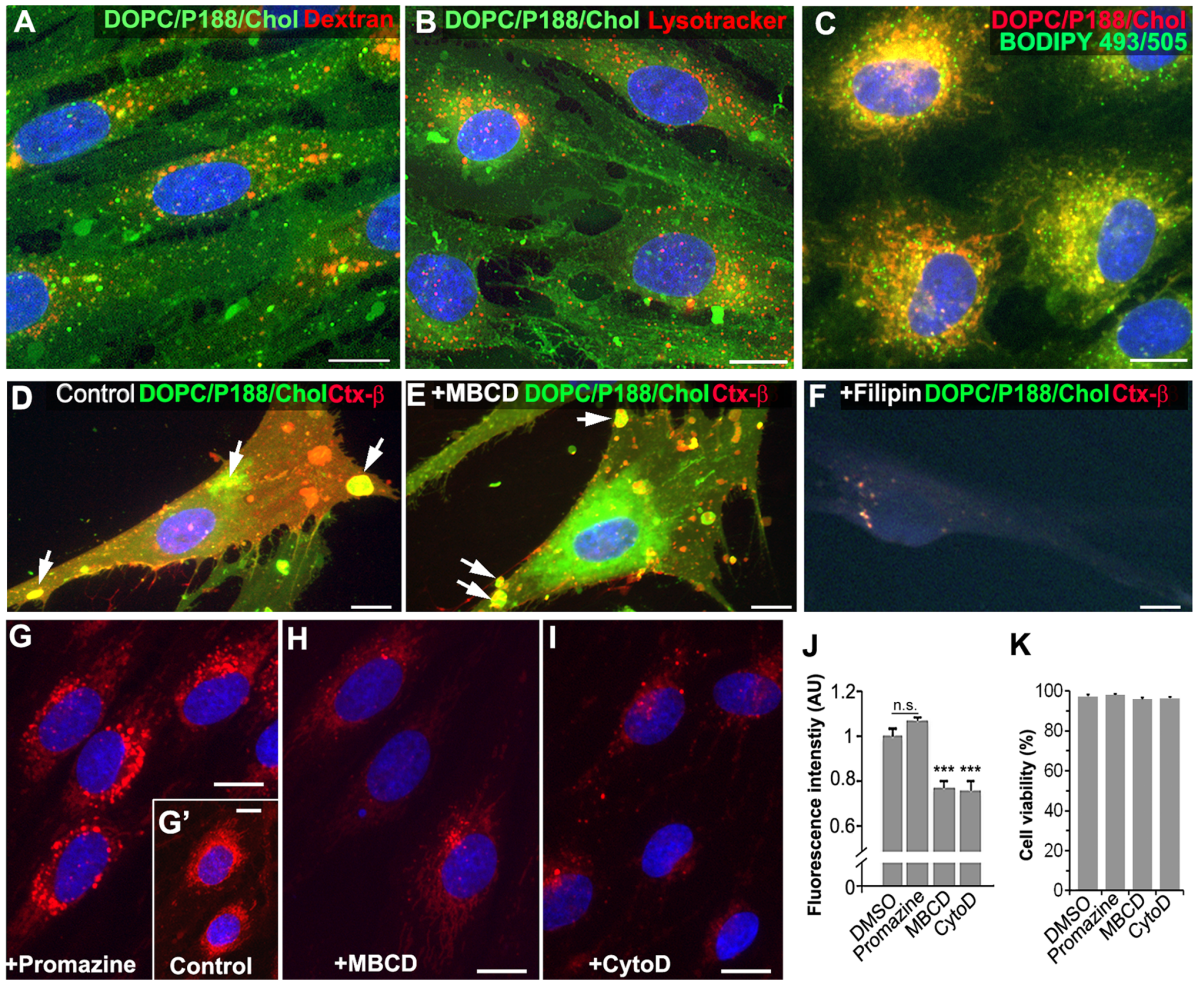
Liposome uptake occurs through non-clathrin mediated mechanisms. Single-plane confocal images of rat Schwann cells incubated with DOPC/P188/Chol liposomes labeled with CF (A, B; D–F; green) or Cy5 (C, G–I; red) are shown. Cells were co-incubated with liposomes and pHrodo-dextran (A), or Lysotracker Red (B), or BODIPY409/503 (green). Cell surface co-labeling of CF-DOPC/P188/Chol liposomes and Ctx-β are displayed (D–F). Cells were labeled without pretreatment (D), or after pretreatment with either methyl-B-cyclodextrin MBCD (E), or filipin (F). Arrows indicate surface-bound liposomes within lipid rafts (D, E). Schwann cells loaded for 8 h with Cy5-labeled liposomes alone (G', inset) or with endocytic inhibitors: promazine (G), MBCD (H), or cytochalasin D; CytoD (I). Nuclei are stained with Hoechst dye, shown in blue (A–I). Scale bars, 10 µm (A–I). Quantification of liposome fluorescence after uptake into Schwann cells (J). Values represent pixel intensity as arbitrary units (AU) +/− SEM. Values were normalized against the DMSO-treated controls. Student's t-test; *** p<0.001; n.s., not significant. Quantification of cell viability by trypan blue dye exclusion assay after treatment with the endocytic inhibitors, as in G–I (K). Each condition was analyzed in triplicates and values represent the averages +/− SEM.

The cellular uptake mechanism for liposomes likely involves endocytosis via clathrin-dependent or -independent pathways. To investigate this, we determined the subcellular localization of DOPC/P188/Chol liposomes within rat Schwann cells after 8 h incubation at 37°C using fluorescent organelle markers (Figure 2). Because of the sensitivity of the fluorescent liposomes to extended fixation and permeabilization required for antibody-mediated staining procedures, all cell type and organelle co-labeling experiments were performed with reagents directly linked with fluorescent markers, rather than with antibodies. As a marker for fluid phase endocytosis (pinocytosis) [21], we loaded red fluorescent dextran (10,000 MW) together with DOPC/P188/Chol liposomes. As shown in the merged single plane confocal image (Figure 2A), only occasional co-localization between dextran (red) and liposomes (green) is observed. Similarly, we found limited co-localization between the lysosomal marker LysoTracker and DOPC/P188/Chol liposomes (Figure 2B) demonstrating that the liposomes are not delivered to degradative compartments. In comparison, Cy5-labeled DOPC/P188/Chol liposomes (red) were extensively co-localized with BODIPY 493/503-marked lipid droplets (green), signifying their association with lipid-rich organelles (Figure 2C).

Next, we tested whether liposomes interact with lipid raft domains, which are enriched with cholesterol, and are active sites of caveloae-dependent endocytosis [22]. Lipid rafts can be identified with fluorescent conjugates of recombinant Cholera toxin subunit β (Ctx-β), which bind to the ganglioside GM1 in lipid rafts [23]. When cells were co-incubated with DOPC/P188/Chol liposomes and Alexa Fluor 594 Ctx-β for 30 min on ice, intense liposome-derived fluorescence is detected within Ctx-β labeled lipid rafts (Figure 2D, arrows). To examine the influence of cholesterol within the plasma membrane on DOPC/P188/Chol liposome binding, we perturbed lipid rafts with a 10 min pre-treatment with methyl-β-cyclodextrin (MBCD), a cholesterol extracting agent. This manipulation alters the binding of liposomes to a more heterogeneous pattern, within aberrant clusters of Ctx-β positive membrane domains (Figure 2E, arrows). In agreement, depletion of cholesterol with a 10 min pre-treatment with filipin (10 μM) almost completely abolishes cell surface liposome binding (Figure 2F).

The association of the DOPC/P188/Chol liposomes with lipid rafts suggests that they undergo internalization through clathrin-independent mechanisms. Using pharmacologic inhibitors of clathrin-dependent and -independent endocytosis, we evaluated the uptake of Cy5-DOPC/P188/Chol liposomes into cultured Schwann cells (Figure 2G–I). Consistent with the minimal co-localization with internalized dextran (Figure 2A), which requires clathrin-mediated mechanisms, liposome uptake is not reduced upon blocking the formation of clathrin-coated pits with promazine (0.5 μM) (Figure 2G). In contrast, disruption of lipid rafts by cholesterol extraction with MBCD (4 mM) during the liposome incubation (Figure 2H), or inhibition of actin polymerization with cytochalasin D (CytoD; 0.1 μg/ml) (Figure 2I), notably diminishes intracellular fluorescence. Indeed, semi-quantitative assessment of fluorescence intensity reveals a highly-significant reduction in liposome uptake in the presence of MBCD or CytoD, as compared to control (Figure 2J, ***p<0.001). To rule out cellular toxicity as a potential reason for decreased liposome internalization during these experiments, we performed trypan blue cell viability studies. As shown in the graph (Figure 2K), incubation of the cells with the named endocytic inhibitors did not lead to a decrease in cell viability. Identical effects on liposome uptake were observed using green fluorescent CF-liposomes (not shown). Together, these results reveal the involvement of lipid raft-dependent endocytosis and actin polymerization (phagocytosis) in the internalization of the liposomes, both of which are clathrin-independent mechanisms.

### Fluorescent liposomes are internalized by neurons and Schwann cells

**Figure 3:**
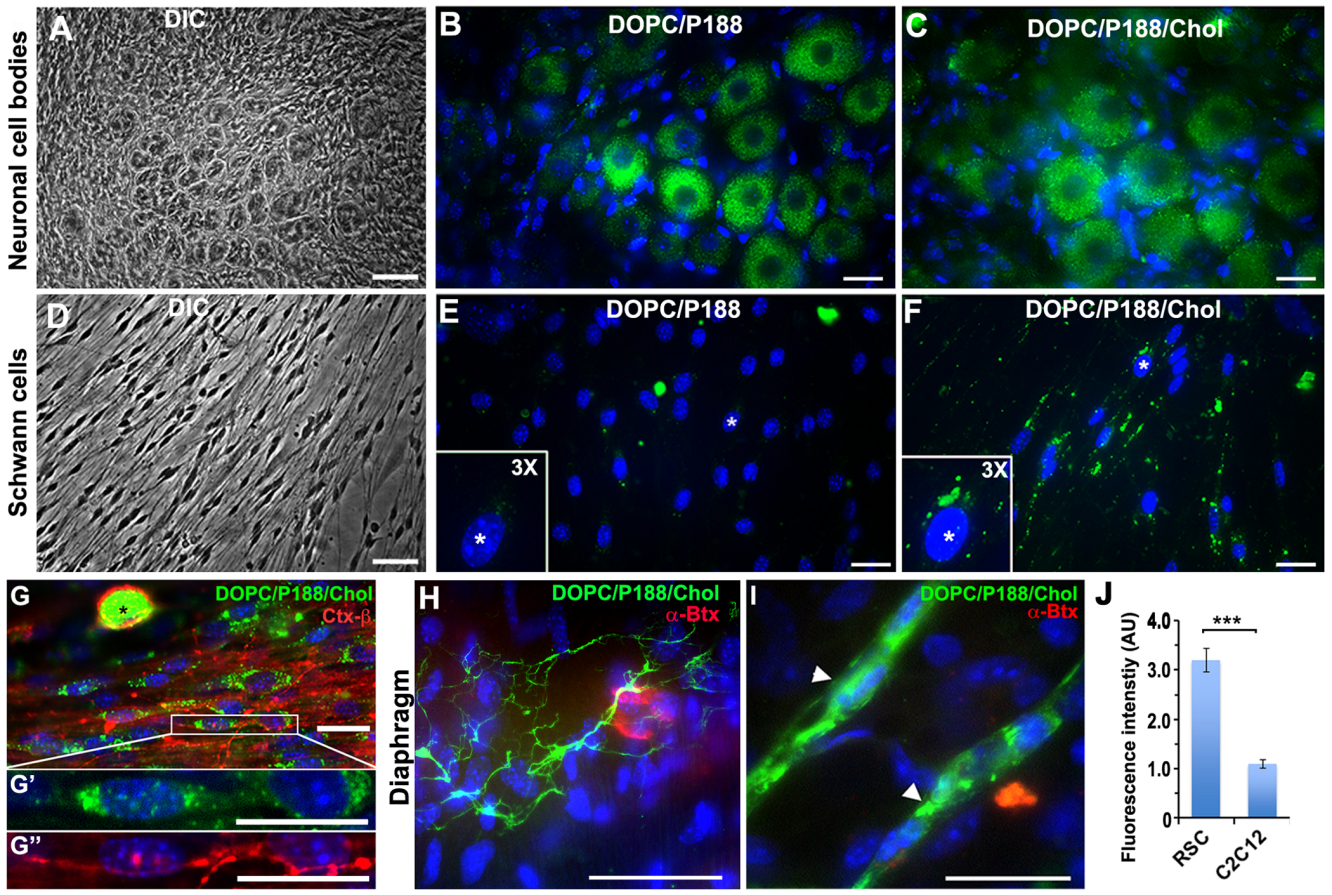
Fluorescent liposomes are internalized by sensory neurons, motor axons and Schwann cells. DRG explants (A–F) after incubation with fluorescent DOPC/P188 (B, E) or DOPC/P188/Chol liposomes (C, F) are shown. Phase contrast images identify clusters of neuronal cell bodies (A) and Schwann cells (D). Insets in E and F represent 3-fold magnification of cells marked with an asterisk (*). Cy5-DOPC/P188/Chol liposomes loaded for 72 h (green) followed by Ctx-β labeling (red) are shown (G). A neuronal cell body is marked with an asterisk (*). Single channel views of DOPC/P188/Chol (G') and Ctx-β (G") of the boxed Schwann cells are enlarged below. Micrograph of a freshly-isolated mouse diaphragm after incubation with DOPC/P188/Chol liposomes (green) and α-Btx (red) is displayed (H and I). Axonal processes (H) and Schwann cells (I, arrowheads) demonstrate internalized liposomes. Nuclei are stained with Hoechst dye (B, C, E, F, G–I). Scale bars, 50 µm (A, D H, and I); 20 µm (B, C, E, F, G, G', G"). Quantification of cell fluorescence after CF liposome uptake in rat Schwann cells (RSC) and differentiated C2C12 myotubes (J). Fluorescence is expressed in arbitrary units (A.U.) and values represent means ± SEM. Student's t-test, *** p<0.001.

As a step toward in vivo application, we investigated the uptake of DOPC/P188/Chol liposomes into specific PNS cell types using mouse dorsal root ganglion (DRG) and diaphragm tissue explants, which contain a mixture of sensory neurons, motor axons and Schwann cells (Figure 3). Since DRG explants lack a blood-nerve barrier, this system allows testing the uptake of liposomes by neurons and glia without prior entry from the vasculature. When DRG explants are exposed to fluorescent DOPC/P188 or DOPC/P188/Chol liposomes, the two formulations show equally effective uptake within sensory neurons (Figure 3B, C), whose clustered cell bodies can be visualized under phase microscopy (Figure 3A). In contrast, liposome uptake by Schwann cells in the periphery of the same explant cultures (Figure 3D) is low for DOPC/P188 (Figure 3E), but is enhanced by the addition of Chol (Figure 3F). Concerning the Schwann cells, these results are consistent with findings in the dissociated glial cultures (Figure 1). It is also of interest that while the SH-SY5Y neurons show a clear preference for the cholesterol containing liposomes (Figure 1A), particle uptake by sensory neurons is not influenced by the presence of this lipid (Figure 3B, C). The comparable uptake of DOPC/P188 and DOPC/P188/chol particles by sensory neurons indicate that cholesterol is not critical for internalization by these cells. Yet, both sensory neurons and myelinating glia contain abundant lipid rafts [24]. To corroborate this observation, we probed the DRG explant cultures with fluorescently labeled Ctx-β after loading with liposomes for 72 h (Figure 3G). Internalized DOPC/P188/Chol liposomes are prominent within the cell body of a Ctx-β-labeled sensory neuron (Figure 3G; asterisk), as well as in elongated Schwann cells (Figure 3G-G").

To further test whether Schwann cells and motor axons can be targeted with liposomes within tissue, we evaluated liposome uptake in freshly-isolated diaphragm explants *ex vivo* [20]. In acute diaphragm preparations from 4–8 week old mice, we observe fluorescent liposome (green) uptake in terminal axonal processes near the vicinity of motor end-plates labeled with α-Btx (red) (Figure 3H). Within the same tissue explant, we also detect internalized liposomes within Schwann cells (Figure 3I, arrowheads), but only negligible uptake in surrounding myofibers (identified by Hoechst nuclear stain). To substantiate the preferential uptake of DOPC/P188/Chol by glial cells as compared to muscle, we evaluated Cy5-DOPC/P188/Chol liposome uptake by Schwann cells and differentiated C2C12 myotubes. After loading for 24 h, Cy5-DOPC/P188/Chol-associated fluorescence in Schwann cells is 3-fold greater than in differentiated C2C12 myotubes (Figure 3 J; *** p<0.001, Students t-test). This result provides further evidence that within neuromuscular tissues, neurons and Schwann cells are more active than muscle cells in internalization of this specific liposome formulation.

### Delivery of liposomes to the PNS *in vivo*

**Figure 4:**
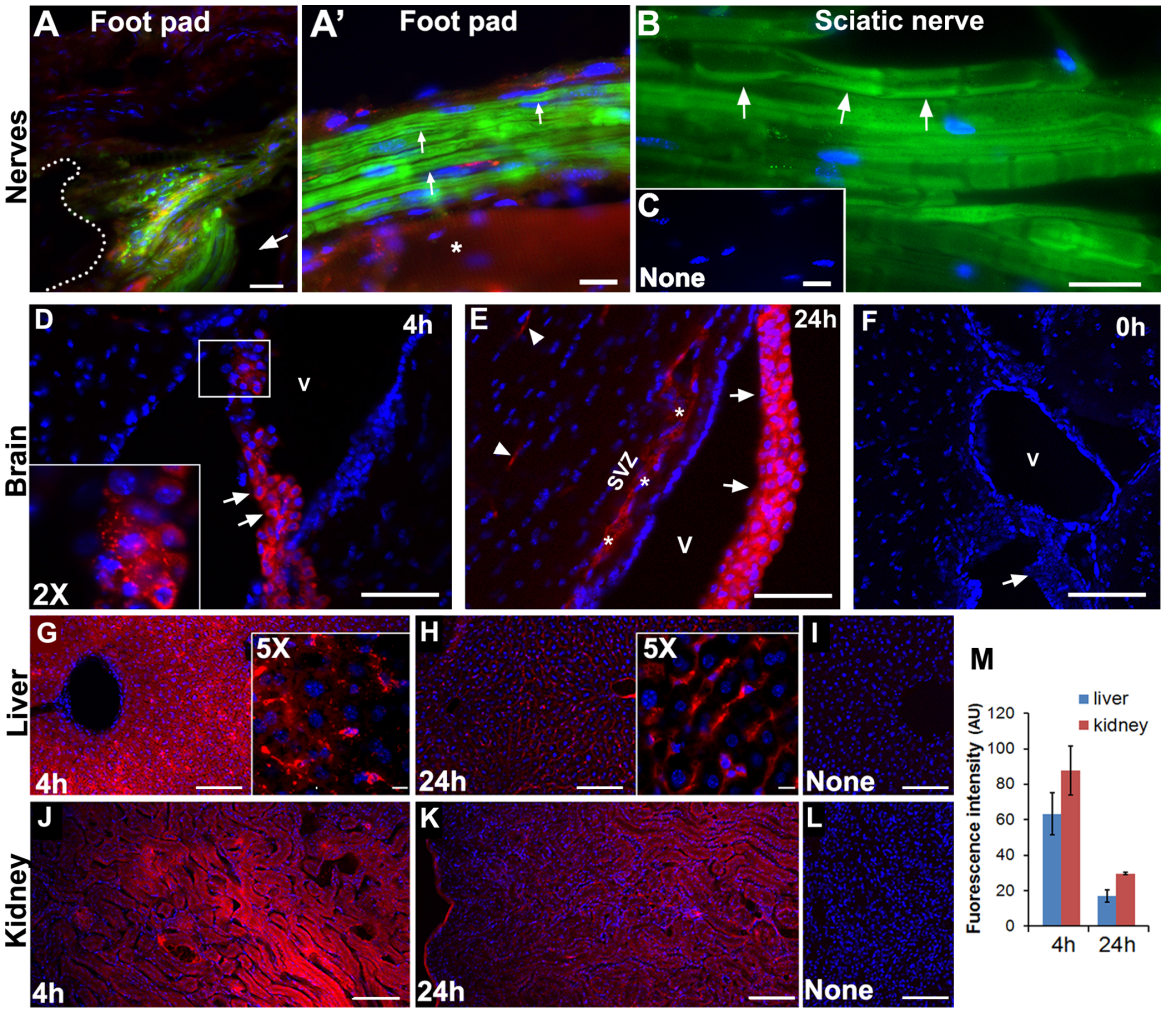
Liposomes administered *in vivo* are detected in peripheral nerves and choroid epithelia. Fluorescent DOPC/P188/Chol liposomes were administered through i.m. (A, A') or i.v. (B–F) injections. Liposome fluorescence is detected in nerves (arrows) near to the injection site of the foot pad (indicated with dotted lines in A). Asterisk in A' marks neighboring muscle tissue. Red fluorescent-tagged dextran was co-administered with liposomes by i.m. injection (A, A'), and labels a distinct subset of cells. Arrows in B indicate liposome-derived fluorescence in myelinating Schwann cells detected after i.v. administration. Non-injected sciatic nerve is shown (C). Images of brain (D–F), liver (G–I) and kidney (J–L) after i.v. injections of Cy5-DOPC/P188/Chol liposomes. Liposome-derived Cy5 fluorescence is identified in the choroid plexus after 4 (D) and 24 h (E) post i.v. injection. Inset in panel D shows a 2X enlarged view of the boxed cells. A control brain (0 h post-injection) is shown in (F). The choroid epithelia (arrows), likely blood vessels (arrowheads), the subventricular zone (SVZ; asterisks) and the lateral ventricles (V) are marked (D–F). Representative images liver (G–I) and kidney (J–L) at 4 and 24 h post-injection, and from uninjected mice are shown. Nuclei are stained with Hoechst dye (blue) (A–L). Scale bars, 20 µm (A–C), 100 µm (D–L). Quantification of liver and kidney tissue-associated fluorescence after 4 and 24 h post i.v. administration of Cy5- DOPC/P188/Chol liposomes (M). Fluorescence is reduced by ∼68% and ∼63% between 4 and 24 h for the liver and kidney, respectively. Fluorescence intensity is expressed in arbitrary units (AU) and values represent means ± SEM.

Liposomes are ideal drug delivery vehicles as they can be administered either by local or intravenous (i.v.) injections. An acute intramuscular (i.m.) injection into the footpad of 2-month old mice results in preferential liposome uptake into myelinated nerves compared to surrounding tissue, when examined 4 h post-injection (Figure 4A, A'). To monitor cellular uptake, we co-injected dextran (red), a retrograde tracer for axonal innervations (Figure 4A and A'). At the site of injection, liposome- and dextran-derived fluorescence prominently labels fibers that resemble myelinated nerves (Figure 4A, arrow). In comparison, surrounding muscle and dermal tissues visualized with Hoechst nuclear dye are not reactive (Figure 4A). This result is in agreement with data obtained in the *ex vivo* diaphragm preparations (Figure 3H and I) where we detect prominent particle uptake by Schwann cells and neurons, but not by surrounding muscle. Under a higher magnification, liposomes administered into the footpad are detected within Schwann cells that display the characteristic “rail-road track” morphology of myelinated nerve fibers (Figure 4A', arrows). Again, liposome-derived fluorescence (green) is absent in underlying muscle tissue (Figure 4A', asterisk). Moreover, the lack of co-localization between liposomes and dextran within the footpad indicates that the particles were likely taken up by distinct mechanisms, similar to the data obtained in cultured Schwann cells (Figure 2A).

To investigate systemic tissue targeting of DOPC/P188/Chol liposomes, we employed tail vein injection in 2-month old mice. As seen with the i.m. injection (Figure 4A), we detect robust liposome fluorescence (green) within myelinated Schwann cells in the sciatic nerve, when examined 4 h post i.v. injection (Figure 4B, arrows). To determine if the liposomes also enter myelinated regions of the CNS, in an independent experiment with Cy5-DOPC/P188/Chol liposomes, we examined the brain following systemic particle administration. Within the lateral ventricles, robust liposome-derived fluorescence (red) is seen in choroid plexus epithelia, at both 4 h and 24 h post injection (Figure 4D and E). At the 24 h time point, we also find liposome-associated fluorescence within the brain parenchyma, likely associated with blood vessels (Figure 4E, arrowheads). The subventricular zone, the region along the walls of the lateral ventricle, is notably reactive for liposomes at the 24 h time point.

Within the same mice, we followed the clearance of the particles through the liver (Figure 4G–I) and kidney (Figure 4J–L). At the 4 h time point, both the liver and kidney display intense Cy5-derived fluorescence (Figure 4G, J), while after 24 h, the signal is notably reduced in both organs (Figure 4H and 4K). Indeed, semi-quantitative analyses of tissue-associated fluorescence indicates an approximately 65% reduction from 4 to 24 h (Figure 4M). Based on the composition of the liposomes, these clearance routes were anticipated as particles with high lipid content are cleared by the liver, while transcytosis through kidney endothelial cells is promoted by poloxamer [25]. Together, these *in vivo* studies indicate that through systemic administration, the described DOPC/P188/Chol liposomes cross the BNB and are preferentially taken up by myelinated peripheral nerves.

## Discussion

The availability of effective delivery vehicles that can reach specific and often distant cells of the PNS would fill an unmet medical need. Peripheral neuropathies associated with acquired medical conditions such as diabetes, or genetic abnormalities (e.g., Charcot-Marie-Tooth neuropathies), represent a large heterogeneous group of disorders for which effective therapies are lacking. By fine-tuning the composition of DOPC-based liposomes, we developed a novel formulation that is preferentially taken up by Schwann cells and neurons of the PNS, as compared to surrounding muscle. The lack of liposome uptake by skeletal muscle tissue indicates that these particles might be suitable drug delivery vehicles for a variety of neuromuscular disorders.

Previous studies with PNS targeting liposomes are limited to immuno-liposomal formulations that use monoclonal antibodies to myelin basic protein as the vector [26]. The liposomes described here provide a major advancement over targeting vector-containing nanoparticles, both in terms of cost and potential side effects. The formulation of DOPC is based on knowledge from cancer biology [18], [19] and was modified to mimic specific properties of neural cell membranes, such as lipid composition. As shown by flow cytometry and optical imaging, the inclusion of cholesterol is critical for maximizing uptake into capillary endothelia and Schwann cells. In contrast, cholesterol is not essential for entry into cultured peripheral sensory neurons in DRG explants, suggesting that the particles are taken up by more than one mechanism. As indicated by our pharmacological studies with isolated Schwann cells, the DOPC/P188/Chol liposome internalization occurs mostly by caveolae and actin-mediated mechanisms (Figure 2J). Thus, in addition to uptake through interactions with caveolae, liposomes may enter neurons by serving as substrates for actin-mediated plasma membrane endocytosis, which is active at synaptic terminals [27]. Moreover, the minimal detection of DOPC/P188 and DOPC/P188/Chol liposomes within fibroblasts or muscle cells indicate that the formulation is not randomly absorbed by all cell types or tissues.

With regard to the route of entry, the uptake into Schwann cells occurs via clathrin-independent endocytosis and leads to subcellular distribution within lipid-rich compartments, rather than lysosomes. The escape from degradative compartments is likely facilitated by entry through the caveolae. Caveolae are known to transport their cargo to neutral caveosomes and enter the endoplasmic reticulum and the cytoplasm, bypassing the degradative lysosomes [28]. The diffuse rather than punctate appearance of the particles within the BMECs as well as in the sciatic nerve supports the notion that these liposomes will be suitable for the delivery and release of therapeutic agents within targeted cells. While we recognize that the limitation of liposomes is their small carrier capacity, a number of biological agents with potential application for neural disorders are suitable candidates, including small moleculesand siRNAs. In fact, liposomes can be developed into multifunctional vehicles for imaging and drug delivery, which would provide a key advancement toward clinical applications [29]. The *in vivo* imaging studies show that the liposomes retain the fluorescent marker for over 24 hours, allowing for extended applications. This property of the formulation is critical for therapeutic applications as ideal particles will support sustained release of the drug molecules [29].

In summary, the particles described here provide a promising delivery reagent for therapy development for neuromuscular disorders, including demyelinating neuropathies. The ability of DOPC/P188/Chol formulation to enter myelinated peripheral nerves through local or systemic administration offers a great advantage over existing formulations.
